# Novel compound heterozygous mutations in *LEP* responsible for obesity in a Chinese family

**DOI:** 10.1016/j.ymgmr.2024.101114

**Published:** 2024-06-29

**Authors:** Hui Li, Guodong Liu, Bei Lu, Xin Zhou

**Affiliations:** a Department of Anesthesiology, Zibo Central Hospital, Binzhou Medical University, 255036 Zibo, China; b Department of Gastroenterology, Zibo Central Hospital, Binzhou Medical University, 255036 Zibo, China; c Department of Nuclear Medicine and Radiotherapy, Zibo Central Hospital, Binzhou Medical University, 255036 Zibo, China

**Keywords:** Obesity, Genetics, LEP, Compound heterogeneous, Novel variant

## Abstract

**Background:**

Early childhood obesity poses a significant global public health challenge, necessitating the identification of treatable causes, particularly congenital leptin deficiencies. Serum leptin level measurement aids in diagnosing these rare contributors, guiding effective management.

**Methods:**

A Chinese family with early-onset obesity underwent LEP mutational screening via direct sequencing. mRNA expression and protein stability patterns of LEP were separately analyzed using qPCR and bioinformatics.

**Results:**

We present a case of a 12.5-year-old girl born to non-obese, non-consanguineous Chinese parents, exhibiting low leptin levels. Leptin gene sequencing revealed novel compound heterozygous mutations in exon 3. RT-PCR analysis showed the mutation didn't affect leptin production. Bioinformatics analysis indicated the variant rendered the leptin protein unstable.

**Conclusion:**

Loss-of-function mutations in *LEP* underlies early-onset obesity in the patient.

## 1 Introduction

Obesity, characterized by excessive body fat accumulation, has attained the status of a global epidemic, as delineated by the World Health Organization (WHO) [1]. This multifaceted disorder involves intricate interactions among environmental, behavioral, and genetic factors. The initiation of genetic studies was motivated by the observed familial clustering in obesity cases. In 1997, within a highly consanguineous Pakistani family, congenital leptin deficiency, an exceptionally rare cause of severe early-onset obesity, was first documented. Two afflicted cousins manifested undetectable serum leptin levels due to a homozygous frameshift mutation in *LEP*, leading to the synthesis of a truncated, un-secreted protein [2]. Subsequently, five additional individuals of Pakistani origin with the identical homozygous mutation were identified. A pivotal moment in obesity genetics ensued with the identification of mutations in *LEP* among severely obese children, subsequently expanding to the discovery of mutations in other related genes [3,4].

The human *LEP* locates on chromosome 7q31.3, and its resultant product is the leptin protein, playing a crucial role in regulating human appetite [5]. Leptin, part of the cytokine family, shares membership with vital regulators like interleukin-6. Synthesized initially as a 167-amino acid immature protein, it undergoes processing, resulting in a mature, functional 146-amino acid protein [6]. It is secreted by adipocytes (fat cells), exhibits a direct relationship with body fat mass and body mass index when present in the bloodstream. It significantly influences diverse physiological processes, including energy metabolism, the endocrine system, and the immune system. By targeting the central nervous system, it modulates food intake, thereby controlling body fat. Beyond its role in fat regulation, leptin also performs essential functions in reproductive organs, mammary glands, the immune system, and bone mineral density [7].

Currently, eighteen mutations in the *LEP* gene have been identified, leading to early onset obesity [2,[8], [9], [10], [11], [12], [13], [14], [15], [16], [17], [18], [19], [20], [21], [22], [23]]. In this study, we recruited a Chinese family with very low serum leptin concentrations and obesity. Novel compound heterozygous mutations in *LEP* (NM_002303.3) are responsible for obesity. Among the heterozygous mutations, c.350G > A (p.Cys117Tyr) represents a recurrent mutation, whereas c.451C > T (p.Gln151X) is a novel nonsense mutation leading to the production of a truncated leptin. Our findings contribute to the broadening of the existing spectrum of known *LEP* mutations.

## 2 Materials and methods

### 2.1 Study samples

**Fig. 1:**
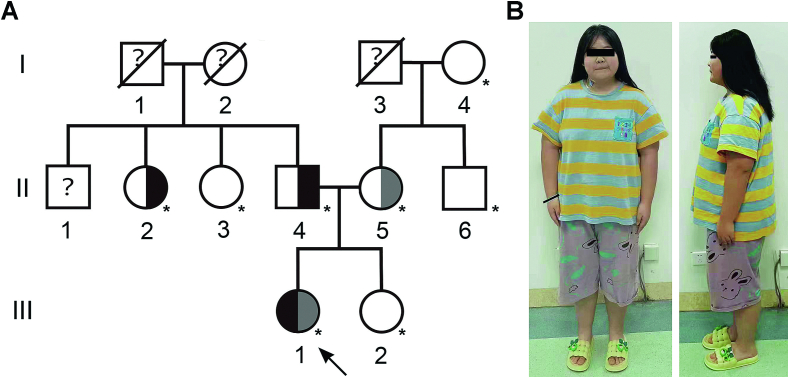
The Chinese family with inherited obesity. (A)Pedigree with obesity caused by *LEP* mutation. Squares and circles indicate males and females, respectively. Half-black or half-dark symbols represent carriers and open symbols represent unaffected individuals. The proband is marked by an arrow. The participants in this study are indicated by an asterisk. (B) Photograph of the patient at the age of 12.5 years.

Blood samples were procured from eight individuals, comprising four unaffected, three carriers, and one affected member, within a small Han Chinese family affected by autosomal recessive obesity (Fig. 1A). The clinical features and graphic data were recorded. All participants provided informed consent, and the study strictly adhered to the ethical principles outlined in the Declaration of Helsinki. Approval for the research protocol was obtained from the Ethics Committee of Zibo Central Hospital.

### 2.2 Mutation screening and sequence analysis

DNA extraction was conducted from 200 μL of peripheral blood using the TIANamp Blood DNA Midi Kit (Tiangen, Beijing, China). Following genomic polymerase chain reaction (PCR), we performed Sanger sequencing on the coding exons and their adjacent intronic sequences of *LEP* genes to identify pathogenic mutations in the proband. The primers utilized in the PCR are detailed in Table 1. Nucleotides and Amino acid conservation analysis around the mutation site was undertaken using the UCSC Genome Browser (http://genome.ucsc.edu/index.html). The stability of wildtype and mutant proteins were predicted by the COREX/BEST server (http://best.bio.jhu.edu/BEST/index.php).

**Table 1:** Primers used for screening mutation in *LEP.*

Exons	Forward (5′ → 3′)	Reverse (5′ → 3′)
Exon 1	CAGTTGCGCAAGTTGTGATC	CAGCTCCCGGTAACCTTCTA
Exon 2	GTCTGGTAATGTGGTTGGTAA	CTGTGCTTTCAAATCCTTCTC
Exon 3–1	AGAGCGATTCCTCCCACATGC	TTACGAGAGAACTAACTGGAG
Exon 3–2	TTGAGTGACTCGAGGGTTGGG	TCTCCACACACCAAACCTTCC
Exon 3–3	AGAGGAGTTTCGAGGTAGAGT	AGCTGACCCCAGTGATGGATG

### 2.3 Quantitative PCR analysis

Total RNA was isolated from peripheral blood cells using the Trizol reagent (Tiangen, China), followed by reverse transcription using the HiScript II 1st Strand cDNA Synthesis Kit (Vazyme, China). Quantitative PCR analysis was carried out on a QuantStudio 5 instrument (Thermo Scientific, USA) with SYBR Green I (Tsingke, China). All samples were analyzed in triplicate and normalized using β-actin. The amplification primers were as follows: LEP (Forward: 5′-TCAATGACATTTCACACACGC-3′, Reverse: 5′-TTGGATAAGGTCAGGATGGGG-3′), and β-actin (Forward: 5′-TCCAGCCTTCCTTCCTGGGCAT-3′, Reverse: 5′-GCACTGTGTTGGCGTACAGGTC-3′).

## 3 Results

### 3.1 Clinical features

We present a case involving a Chinese girl with a body mass index (BMI) of 33.5 kg/m^2^. The anthropometric and endocrinologic characteristics of the proband are summarized in Table 2. At the age of 12.5 years, she measured 156.4 cm in height and weighed 81.9 kg. A clinical examination revealed normal findings, with the exception of hyperphagia and aggressive behavior related to food demands. Notably, her leptin level was notably low at 0.5 ng/mL, with the normal range being 2.0–5.6 ng/mL.

**Table 2:** The physical, hormonal, and metabolic traits of the affected family.

Variables		Reference range
Age (yr)	12.5	
Weight (kg)	81.9	
Height (cm)	156.4	
BMI (kg/m^2^)	33.5	
Body fat (%)	37.7	
Leptin (ng/mL)	0.5	2.0–5.6
Adiponectin (mg/mL)	6.0	5.0–7.5
Total cholesterols (mM/L)	204	<200
Triglycerides (mM/L)	231	<203
AST (U/L)	38	0–40
ALT (U/L)	68	0–40
Thyrotropin (U/L)	1.32	0.53–3.59
TSH (μU/mL)	3.11	2–10
Cortisol (nmol/L)	350	193–690
Insulin (μIU/mL)	27	2.6–40

ALT, Alanine aminotransferase; AST, aspartate aminotransferase; TSH, Thyroid Stimulating Hormone.

Further clinical analysis showed normal levels of Adiponectin, AST, ALT, Thyrotropin, thyroid-stimulating hormone (TSH), and cortisol. The insulin profile was also within the normal range. Crucially, her parents are two healthy, nonobese Chinese individuals without any known consanguinity.

### 3.2 Genetics

**Fig. 2:**
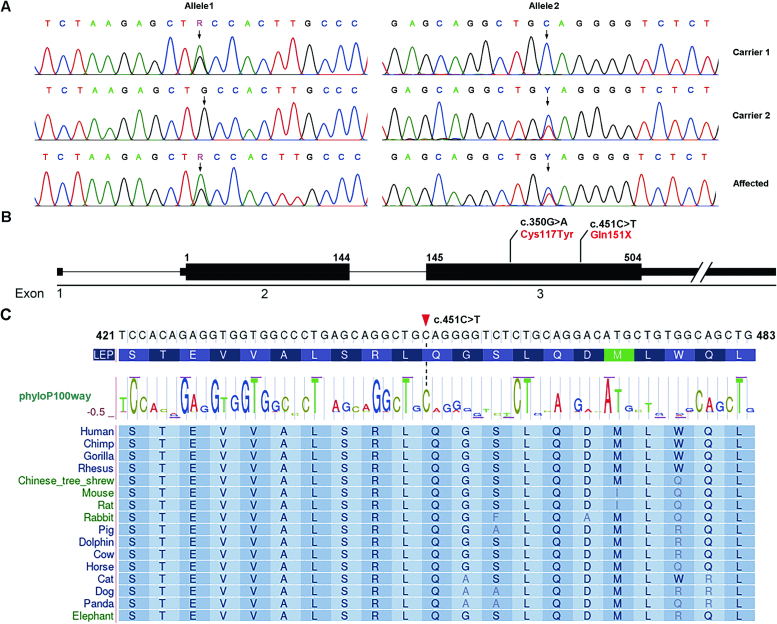
Compound heterozygous mutations of *LEP* in the family with obesity. (A) Sequence chromatogram showing the compound heterozygous mutations (c.350G > A and c.451C > T) of LEP in the proband. The mutation was numbered according to GenBank NM_000230. (B) The positions of LEP mutations are marked in exon 3. (C) Nucleotides and amino acids alignment of mammalian samples show that the regions around the novel nonsense mutation are highly conserved. Numbers on the left and right indicate the position of this fragment, with the mutant's position marked by a red triangle.

Sequencing the *LEP* gene revealed a recurrent missense mutation (c.350G > A, p.Cys117Tyr) and a novel nonsense mutation (c.451C > T) in exon 3 of the *LEP* gene in the proband. Carriers exhibit an affected status with a single allele mutation in the *LEP* gene (Fig. 2A, B). The novel variant resulted in the substitution of a newly formed stop codon for a phylogenetically conserved glutamine residue (p.Gln151X) (Fig. 2C). Apart from a few nonpathogenic SNPs, no other variants were identified.

### 3.3 qPCR analysis of *LEP* mRNA in the family

The pathogenicity of the recurrent missense mutation, c.350G > A, has been confirmed in previous reports [8]. The novel variant, c.451C > T, signifies a nonsense mutation, implying a potential influence on *LEP* mRNA decay through the nonsense-mediated decay pathway, rather than the production of a truncated protein. To elucidate the effects of this novel variant, we conducted qPCR to assess *LEP* mRNA levels in the family.

**Fig. 3:**
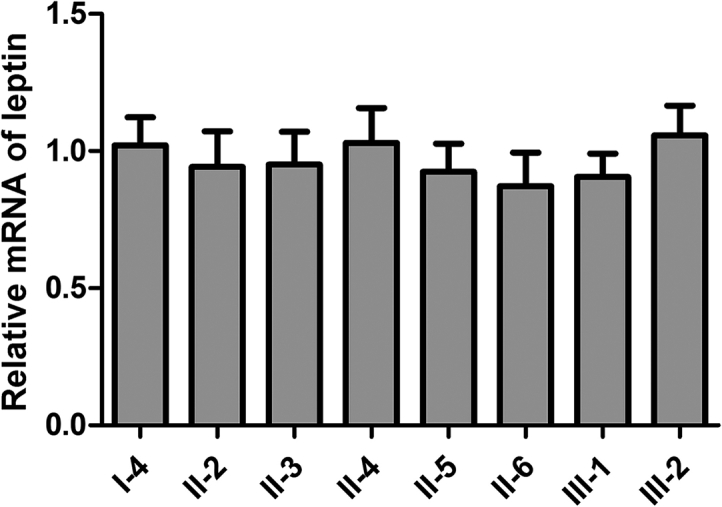
qPCR analysis of *LEP* mRNA in the family. The peripheral blood cells with wild-type (WT) or mutant (c.451C > T) show a similar relative LEP mRNA expression level. All samples were analyzed in four replicates and β-actin was used as internal control.

The results showed no significant difference in the mRNA levels of *LEP* among unaffected individuals, carriers, and affected individuals (Fig. 3). This suggests a higher likelihood for the novel variant to produce a truncated protein instead of initiating RNA decay.

### 3.4 Bioinformatics analysis of leptin mutants

**Fig. 4:**
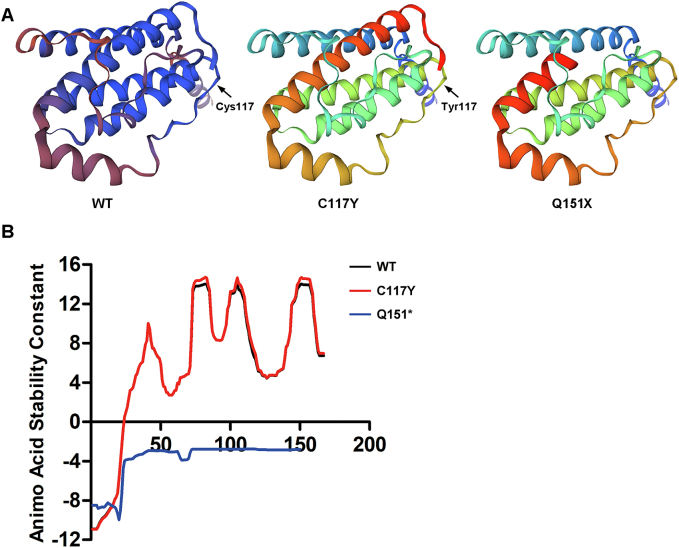
Bioinformatics analysis of the leptin mutants. (A) The predicted 3D structure shows no difference between wildtype and the mutants. (B) The residue-specific stability constant for each protein residue was predicted using the BEST/COREX server.

The mutant Q51X (c.451C > T, p.Gln151X) undergoes a truncation of 17 amino acids at the C-terminus in comparison to the wild type. Importantly, the mutation does not impact the main 3D structure of the protein. Similarly, the mutant C117Y (c.350G > A, p.Cys117Tyr) exhibits a structure closely resembling that of the wild type (Fig. 4A). These findings strongly indicate that the pathogenicity associated with these mutations does not arise from alterations in the protein's structural conformation. The integrity of the main structural framework suggests that factors other than changes in protein structure may contribute to the observed pathogenic effects.

To further investigate the underlying mechanisms, we analyzed the stability of the mutant leptin. The mutant C117Y has nearly the same stability constant as the wild type. However, compared to the wild type, the mutant Q51X is highly unstable (Fig. 4B), undergoing rapid degradation. Our results suggest that an inadequate quantity of leptin is responsible for obesity of the patient.

## 4 Discussion

Obesity primarily stems from imbalances in regulating food intake and energy expenditure, disrupting signaling pathways governing complex body systems like hunger, lipid metabolism, and sugar metabolism [24]. While various syndromes are linked to obesity, the literature also highlights several monogenic forms, with the most common resulting from pathogenic variants in the leptin–melanocortin pathway [25]. Notably, congenital leptin deficiency, the sole causally treatable form among all monogenic obesity conditions, is inherited in an autosomal recessive manner [26]. In our present study, we identified compound heterozygous mutations (c.350G > A, c.451C > T) in the *LEP* gene within an obesity-related family, exhibiting a loss-of-function feature.

Leptin, a hormone released by adipose tissue, is intricately linked to adiposity in humans [27]. Its expression is constant, and during prolonged caloric deficits, both fat stores and leptin production decrease [28]. Operating as a cytokine, leptin is pivotal in regulating energy balance by influencing feeding behavior and energy expenditure. Recognized as an anorexigenic hormone, it emerges as a primary indicator of adiposity and a signal for nutritional status, with plasma levels showing a robust correlation with adipocyte count and fat mass [29].

Recent research, incorporating exome sequencing, has expanded the inventory of known variants, yet *LEP* mutations continue to be exceedingly rare. Presently, eighteen distinct mutations in the *LEP* gene have been documented as contributors to severe obesity [2,[8], [9], [10], [11], [12], [13], [14], [15], [16], [17], [18], [19], [20], [21], [22], [23]] (Table. 3). While the majority of mutations are found on exon 3 of the *LEP* gene, there are no specific mutation hotspots. This complicates the screening process in clinical settings. Compound heterozygotes mutations are particularly challenging to detect due to their subtlety, although they were identified in the patient.

**Table 3:** Summary of Obesity-Related Genetic Variations in *LEP.*

	Nucleotide change	Type of mutation	Amino acid change	References
1	c.1-44del42	Deletion	Not Available	[8]
2	c.34delC	Frameshift	p.Leu12PhefsX59	[9]
3	c.104 T > G	Missense	p.Ile35Ser	[9,10]
4	c.104_106delTCA	In-frame deletion	p.Ile35del	[11]
5	c.163C > T	Nonsense	p.Gln55X	[12]
6	c.215 T > C	Missense	p.Leu72Ser	[13]
7	c.298G > A	Missense	p.Asp100Asn	[14]
8	c.298G > T	Missense	p.Asp100Tyr	[15]
9	c.309C > A	Missense	p.Asn103Lys	[10,16,17]
10	c.313C > T	Missense	p.Arg105Trp	[18]
11	c.350G > A	Missense	p.Cys117Tyr	[8] This study
12	c.350G > T	Missense	p.Cys117Phe	[19]
13	c.353 A > T	Missense	p.His118Leu	[20]
14	c.362G > A	Nonsense	p.Trp121X	[21]
15	c.397_399delGGT	In-frame deletion	p.Gly133del	[22]
16	c.398delG	Frameshift	p.Trp133ValfsX15	[2]
17	c.422C > G	Missense	p.Ser141Cys	[23]
18	c.451C > T	Nonsense	p.Gln151X	This study
19	c.481_482delCT	Frameshift	p.Leu161GlyfsX10	[11]

In our pedigree analysis, these mutations exhibited a recessive inheritance pattern, consistent with the pathogenic characteristics of *LEP*. The obesity-associated mutations in LEP are loss-of-function mutations, impacting both alleles. Among the two variants, c.350G > A (p.Cys117Tyr) is a recurrent mutation with well-documented pathogenicity, while c.451C > T is a nonsense mutation. Cytidine 451 is highly conserved (Fig. 2C) and is not present in unaffected individuals. The nonsense mutation destabilizes LEP (Fig. 4B), potentially leading to its degradation. Additionally, there is a reported mutation, c.481_482delCT, which results in a truncated leptin after amino acid 161. In our study, the nonsense mutation occurs earlier than this reported mutation, resulting in an even shorter truncated protein, suggesting significant impairment of leptin function. Although the mutations were confirmed by Sanger sequencing, next-generation sequencing (NGS) technology remains a highly effective method for the clinical screening of genetic diseases and should be prioritized for adoption [30].

In this study, the stability of mutant leptin was predicted using the COREX/BEST server. Developed in 2005, this server is widely used to predict stability variations within protein structures [[31], [32], [33]]. It achieves this by calculating at the resolution of individual residues and then mapping these variations onto the protein structure. Therefore, the accuracy of the protein structure is critical. Inaccuracies in the protein model can significantly reduce the reliability of predictions, particularly for nonsense mutants. Although the prediction of Q151* suggests instability (Fig. 4B), further experiments are required to validate these findings.

Despite leptin deficiency, the patient demonstrated a notably lower degree of obesity, highlighting the heterogeneity of obesity in clinical contexts. Interventions for obesity, especially through dietary control, are feasible to some extent. Notably, the patient exhibited a remarkably low daily energy intake, suggesting that effective control of energy intake is achievable despite leptin deficiency, thereby reducing the risk of extreme obesity. Additionally, variations in genetic backgrounds among patients may contribute to the increased resistance to the effects of leptin deficiency observed in our case, aligning with findings from experiments in mice [34]. Nevertheless, this significantly heightens the difficulty in diagnosing obesity-related diseases.

It is evident that loss-of-function mutations in *LEP* are implicated in obesity in our research. However, the specific molecular mechanisms underlying this association still require elucidation. Currently, how to effectively identify obesity and provide successful treatment remains an extremely challenging issue.

## Author contributions

Conceived and designed the experiments: Xin Zhou. Performed the experiments: Hui Li, Guodong Liu. Analyzed the data: Bei Lu, Guodong Liu. Contributed reagents/materials/analysis tools: Hui Li. Wrote the paper: Xin Zhou.

## CRediT authorship contribution statement

**Hui Li:** Data curation. **Guodong Liu:** Resources, Data curation. **Bei Lu:** Software, Investigation. **Xin Zhou:** Writing – original draft, Project administration.

## Declaration of competing interest

The authors have declared that no competing interests exist.

## Data Availability

Data will be made available on request.
